# Effects of a mouthwash with chlorine dioxide on oral malodor and salivary bacteria: a randomized placebo-controlled 7-day trial

**DOI:** 10.1186/1745-6215-11-14

**Published:** 2010-02-12

**Authors:** Kayoko Shinada, Masayuki Ueno, Chisato Konishi, Sachiko Takehara, Sayaka Yokoyama, Takashi Zaitsu, Mari Ohnuki, Fredrick Allan Clive Wright, Yoko Kawaguchi

**Affiliations:** 1 Department of Oral Health Promotion, Graduate School of Medical and Dental Sciences, Tokyo Medical and Dental University, Japan; 2 Centre for Oral Health Strategy, NSW, Australia

## Abstract

**Background:**

Previous research has shown the oxidizing properties and microbiological efficacies of chlorine dioxide (ClO_2_). Its clinical efficacies on oral malodor have been evaluated and reported only in short duration trials, moreover, no clinical studies have investigated its microbiological efficacies on periodontal and malodorous bacteria. Thus, the aim of this study was to assess the inhibitory effects of a mouthwash containing ClO_2 _used for 7 days on morning oral malodor and on salivary periodontal and malodorous bacteria.

**Methods/Design:**

A randomized, double blind, crossover, placebo-controlled trial was conducted among 15 healthy male volunteers, who were divided into 2 groups. Subjects were instructed to rinse with the experimental mouthwash containing ClO_2 _or the placebo mouthwash, without ClO_2_, twice per day for 7 days. After a one week washout period, each group then used the opposite mouthwash for 7 days. At baseline and after 7 days, oral malodor was evaluated with Organoleptic measurement (OM), and analyzed the concentrations of hydrogen sulfide (H_2_S), methyl mercaptan (CH_3_SH) and dimethyl sulfide ((CH_3_)_2_S), the main VSCs of human oral malodor, were assessed by gas chromatography (GC). Clinical outcome variables included plaque and gingival indices, and tongue coating index. The samples of saliva were microbiologically investigated. Quantitative and qualitative analyses were performed using the polymerase chain reaction-Invader method.

**Results and Discussion:**

The baseline oral condition in healthy subjects in the 2 groups did not differ significantly. After rinsing with the mouthwash containing ClO_2 _for 7 days, morning bad breath decreased as measured by the OM and reduced the concentrations of H_2_S, CH_3_SH and (CH_3_)_2_S measured by GC, were found. Moreover ClO_2 _mouthwash used over a 7-day period appeared effective in reducing plaque, tongue coating accumulation and the counts of *Fusobacterium nucleatum *in saliva. Future research is needed to examine long-term effects, as well as effects on periodontal diseases and plaque accumulation in a well-defined sample of halitosis patients and broader population samples.

**Trial registration:**

ClinicalTrials.gov NCT00748943

## Background

The mouth is home to hundreds of bacterial species that produce several fetid substances as a result of protein degradation [1]. Oral malodor, also called halitosis or bad breath, is a general term used to describe an offensive odor emanating from the oral cavity. It is caused by several factors [2]. Although some extraoral condition (nasal inflammation, diabetes mellitus, uremia, etc.) have been suggested causes of oral malodor, clinical studies have shown that intraoral causes such as gingivitis, periodontitis and tongue coating are the main sources of the disorder [3,4]. In particular, it has been found that periodontal bacteria produce several malodorous compounds such as volatile sulfur compounds (VSCs). Most of the major compounds contributing to oral malodor are VSCs such as hydrogen sulfide (H_2_S), methyl mercaptan (CH_3_SH) and dimethyl sulfide ((CH_3_)_2_S). The substrates for VSCs are largely sulfur-containing amino acids (i.e. cysteine, cystine and methionine) that are found in saliva, gingival crevicular fluid and tongue coating debris [5].

*In vitro *studies have demonstrated that oral gram-negative anaerobic bacteria such as *Prophyromonas gingivalis (P.g.), Fusobacterium nucleatum (F.n.), Tannerella forsythensis (T.f.), Treponema denticola (T.d.)*, and several species of other oral bacteria can produce VSCs [6]. These bacteria can be isolated from the subgingival plaque in gingivitis or periodontitis patients, and from the saliva and the dorsum of the tongue in healthy subjects [7,8]. Odor outcomes are significantly correlated with total counts of bacteria and higher numbers and proportions of certain gram-negative anaerobes, in particular *F.n*. [9]. *Prophyromonas gingivalis, F. n., and T.f*., which are gram-negative anaerobic rods, actively produce VSCs such as H_2_S and CH_3_SH. *Treponema denticola*, which is a helical microorganism, also produces VSCs [10].

Antibacterial agents such as chlorhexidine (CHX), cetylpyridinium chloride (CPC), triclosan, essential oils, zinc salts, hydrogen peroxide, sodium bicarbonate and chlorine dioxide (ClO_2_) have been tested, either alone or in different combinations with mechanical devices, for their efficacy to reduce oral malodor [11]. CHX being the most studied antimicrobial agent has also been tested for its efficacy in the treatment of oral malodor. Results from a case series study in halitosis patients suggested a significant effect of 0.20% or 0.12% CHX rinsing [12,13]. Although CHX is considered the most effective oral antiseptic agent, the use of CHX for extended periods of time is related to some side-effects, such as tooth and tongue staining, bad taste and reduced taste sensation [14,15].

Previous studies have suggested that ClO_2 _and the chlorite anion (ClO_2_^-^) directly oxidize VSCs to non-malodorous products and, through this oxidation, consume the amino acids such as cysteine and methionine, which act as precursors to VSCs [16]. Moreover, the chlorite anion is powerfully bactericidal to microorganisms [17-19]. Recently a mouthwash containing ClO_2 _has become available on the Japanese market (ClO2 Fresh^®^, Bio-Cide International, Inc., Oklahoma, USA and Pine Medical Co., Tokyo, Japan). Shinada, et al. reported that the ClO_2 _mouthwash was effective at reducing morning oral malodor for 4 hours when used by healthy subjects [20]. Its clinical efficacies on oral malodor have been evaluated and reported only in short duration (maximum 96 h) trials [21,22]. Moreover, no clinical study has investigated its microbiological efficacy on periodontal and malodorous bacteria.

Some mouthwash trials of healthy subjects reported that a significant positive correlation was observed between the reduction in salivary and tongue coating bacteria and oral malodor [8,23]. In this study, because of the difficulties in standardizing tongue dorsum samples, we monitored only the salivary bacteria.

Because the effective antimicrobial action of a mouthwash containing ClO_2_has been verified *in vitro *[16], the hypothesis tested in this study is that a ClO_2 _mouthwash will also effectively reduce oral malodor and periodontal and malodorous bacteria in saliva. Thus, the aim of this study was to assess the inhibitory effects of a mouthwash containing ClO_2 _used for 7 days on morning oral malodor and on salivary periodontal and malodorous bacteria.

## Methods

### Subjects

Subjects were 15 healthy male volunteers aged 19-38 years (mean age 22.9 ± 6.2 years) who had no medical disorders, were not undergoing antibiotic or other antimicrobial therapy, and were non-smokers. The subjects received verbal and written information about the study and signed consent forms to participate. An oral examination was conducted to assess oral status of the subjects prior to the experiment. We excluded females because their menstrual cycle might affect oral malodor on the crossover design with one week washout [24]. No subjects reported usually using commercial mouthwash, antibacterial tooth paste, tongue brush and dental floss.

All dental examinations were conducted by the one trained examiner for all subjects, both for baseline and for follow-up examinations. The examiner was blind to the assignment of subjects to Experimental or Placebo groups.

The sample size was estimated using an expected mean organoleptic measurement (OM) score difference of 1, a within-subject variance around the mean OM score difference of 0.5, a significance level of 5%, and a power of 80%. The results indicated a required sample size of 15 subjects for a crossover design.

### Study design

This clinical trial was a randomized, double blind and crossover design with a one week washout period between the crossover phases. The subjects were randomly assigned to two groups using computer-generated random numbers. Allocation of subjects was generated by one person who was not related with the researchers or subjects of this study, using a random number table. Assignment of subjects was held in a secure and sealed file and only decoded at the end of this trial.

In the first test phase, the group 1 subjects (N = 8) were instructed to rinse with 10 ml of the experimental mouthwash containing ClO_2 _for 30 seconds twice per day (after waking and before sleeping) for 7 days, and those in group 2 (N = 7) to rinse with the placebo mouthwash without ClO_2_. In the second test phase, after a one week washout period, each group then used the opposite mouthwash for 7 days. At baseline, and after 7 days, oral malodor was evaluated by the organoleptic measurement (OM) and gas chromatographic (GC) methods. Concentrations of H_2_S, CH_3_SH and (CH_3_)_2_S were evaluated with GC. Clinical outcome variables included plaque and gingival indices [25,26], tongue coating index (TCI) and tongue discoloration index (TDI) [27,28]. Subjects continued their usual oral hygiene practices during the study, except for the morning of each clinical assessment.

Samples of the resting whole saliva were collected into a sterile plastic tube over a 5 minutes period, prior to oral status assessments, and were immediately sent to the BML dental laboratory (BML Inc., Saitama, Japan). They were microbiologically investigated by an Invader PLUS technology which was applied to detect the presence and quantification of *F.n., P.g., T.f*. and *T.d*. [29].

There were no significant differences in any malodor measures between the two groups at either the first or second baseline measurements before mouth rinsing. Similarly, there were no significant differences between the first and second baseline measurements after one week washout period. Hence, we concluded that the oral condition returned to baseline during the one week washout period, and that one week washout was sufficiently long enough for an explorative trial such as this short trial for 7 days.

Subjects reported their perception of any side effects or compliance issues in rinsing for 7 days with both mouthwashes, and the time schedules they used for the mouth rinsing over the 7 day period. The experimental (with ClO_2_) and the placebo mouthwashes (without ClO_2_) were prepared by Pine Medical Co. for this study [20]. Neither the examiner nor the subject knew whether the mouthwash was experimental or placebo. The contents of each mouthwash were as follows. The experimental mouthwash (ClO2 Fresh^®^) contained 0.16% (w/w) sodium chlorite (NaClO_2_) with an efficacy of 0.10% (w/w) chlorine dioxide (ClO_2_), glycerin, mint oil, 1.13% (w/w) citric acid (a pH adjusting agent) and distilled water, the pH was 5.65, and oxidation-reduction potential (ORP) 588 mv. The placebo mouthwash contained glycerin, mint oil and distilled water; essentially the same contents as those in the experimental mouthwash except for the ClO_2_, with a pH5.83, and ORP of 610 mv. Both mouthwashes were thoroughly membrane filtered and put into plastic bottles sealed with a screw-cap. An independent person, outside this trial filled white, opaque bottles, coded either A or B with the Placebo or Experimental solution. Neither examiners nor subjects in the research group knew which were the Experimental or Placebo solution until the trial was completed.

### Oral malodor assessments

Measurements were conducted at around 9 am to evaluate morning breath odor. Subjects were instructed to abstain from eating strong-smelling foods for at least 48 hours, from using scented cosmetics for 24 hours and from drinking alcohol for 18 hours before the assessment. In addition, they were advised not to ingest any food or drink, and to omit their usual oral hygiene practice on the morning of the assessment day [8]. Oral malodor was evaluated before rinsing (at baseline) and after using the mouthwash for 7 days.

#### Organoleptic measurement (OM)

The OM score was measured by two trained judges after subjects closed their mouth for 3 minutes. Judges were asked to rate malodor on a 0-5 scale, where a score of 0 = represented absence of odor, 1 = barely noticeable odor, 2 = slight malodor, 3 = moderate malodor, 4 = strong malodor and 5 = severe malodor [30]. If two judges gave different scores a mean score was used as the representative score for the subject. The inter-examiner reliability, using Cohen's kappa test, was 0.72-0.76.

#### Gas chromatography (GC) analysis

The GC analysis was carried out using a GC8A gas chromatograph (Shimadzu Co., Kyoto, Japan), equipped with a flame photometric detector. After subjects closed their mouth for 3 minutes, the Teflon^® ^tube connected to the auto-injector was gently inserted into the center of the oral cavity through the lips and teeth, while the lips remained closed. Following aspiration of 20 mL of mouth air with a syringe connected to the outlet of the auto-injector, a 10 mL sample of air was transferred to the column and chromatograph [31]. A sulfur chemiluminescence detector that specifically responds to sulfur was used. VSCs were identified by characteristic retention times and were quantified via comparison of their peak area with that of dilutions of standards. Standard gases of H_2_S, CH_3_SH and (CH_3_)_2_S were prepared with a PD-1B permeater (Gastec Co., Kanagawa, Japan). Before the assessment, the ambient air was used for a baseline calibration.

### Oral status assessments

#### Plaque index (PI) and Gingival index (GI)

The clinical assessments of PI [25] and GI [26] were performed on the four sites (buccal, lingual, mesial and distal) of the six key teeth (FDI tooth number; 16, 12, 24, 36, 32, 44) [32]. Each of the sites is given a score from 0-3 depending on the severity of the periodontal or gingival condition. All measurements were performed by the same examiner, who was blind to which mouthwash was used.

#### Tongue coating index (TCI)

The original index was proposed by Winkel and used by Gómez [27,28]. The index was modified slightly by dividing, the dorsum of the tongue, into nine sections. The dorsum of the tongue is firstly divided into three parts: a posterior, a middle and an anterior part. Sub division is then made with middle and two lateral areas for each of the posterior, middle and anterior thirds of the tongue. Presence of tongue coating is recorded for each of these sections, provided the coating is covering more than 1/3 of each section. The tongue coating in each of the nine sites was scored: 0 = no coating; 1 = light coating; and 2 = thick coating. The "light" category was a thin tongue coating with the papillae clearly visible; and the "thick" category was assigned when a dense coating totally obscured the papillae and they were not visible [33]. The tongue coating value was obtained by the addition of all nine scores, giving a range 0-18. The scores were measured by one trained examiner.

#### Tongue discoloration index (TDI)

In the nine sites of the tongue, each site was scored: 0 = no discoloration; 1 = light discoloration; and 2 = severe discoloration [27,28]. The tongue discoloration value was obtained by the addition of all nine scores, range 0-18. The scores were measured by one trained examiner.

### Microbiological study

The microbiological samples were collected and processed according to a strict protocol within a commercial laboratory (BML Inc.: Dental Laboratory, Saitama, Japan). The modified Invader PLUS technology was used to detect the presence of *F.n*., *P.g., T.f*., and *T.d*. in the resting saliva of each subject at baseline and after 7 days [29].

Nucleic acids were extracted from bacteria colonies of *F.n*.(ATCC 25586), *P.g*(ATCC 33277) and *T.d*.(ATCC 35404) with a silica column and a commercial kit (QlAamp DNA mini kit; QIAGEN, Hiden, Germany). The DNA concentrations of these bacteria and of *T.f*.(ATCC 43037D) were measured by PicoGreen assay (Invitrogen, Carlsbad, CA, USA). Primary probes and Invader oligos were designed with the Invader technology creator (Third Wave Technologies, Madison, WI, USA) and were based on sequences in the amplified regions [29]. Template DNA was added to a 15-μl reaction mixture containing primers for each species, 50 μM deoxynucleotide triphosphate, 700 nM primary probe, 70 nM Invader oligo, 2.5 U PCR enzyme (AmpliTaq Stoffel fragment, Applied Biosystems, Foster City, CA, USA), and Invader core reagent kit (Cleavase XI Invader core reagent kit, genomic DNA, TWT), which consisted of fluorescence resonance energy transfer mix and enzyme/MgCl_2 _solution [29]. The reaction mixture was preheated at 95°C for 2 min, and a two-step PCR reaction was carried out for 35 cycles (95°C for 1 sec, 63°C for 1 min) in the real-time PCR system (LightCycler 480, Roche Diagnostics, Basel, Switzerland). The fluorescence values of carboxyfluorescein (FAM) (wavelength/bandwidth: excitation, 485/20 nm; emission, 530/25 nm) were measured at the end of the incubation/extension step at 63°C for each cycle.

The limit of detection of the m-Invader PLUS was determined for each species with dilutions of bacterial DNA. Standard curves were made based on the crossing point determined by fit point methods [29].

### Subjects perceptions

Subjects reported their perceptions of any side effects or compliance issues in rinsing over the 7 days with both mouthwashes, and the time schedules of rinsing over the 7-day period was recorded in a standard logbook format.

### Statistical Analysis

Statistical analysis was performed using the software program Statistical Package for Social Science (SPSS 15.0J). Means and standard deviations of the clinical indices were calculated, following which the oral examination scores between the two mouthwashes were compared by the Mann-Whitney *U*-test. The differences of the oral examination scores between, before and after rinsing at baseline and after 7 days use of the mouthwash were analyzed with the Wilcoxon signed-rank test. The concentrations of VSCs were log transformed before statistical analysis to make VSCs values approximate normal distribution. Therefore the log transformed concentrations of the VSCs between the two mouthwashes were compared by independent Student's t-test, and those between before and after rinsing at baseline and after 7 days were analyzed with paired t-test. Bacterial counts were log transformed before statistical analysis. Because the detection threshold for this method was 10 copies of bacterial DNA per reaction, we change the bacterial number of not-detected samples to the detection threshold, 10 copies and were log transformed. The mean number counts were calculated using the data of 15 subjects. Data were analyzed using analysis of covariance (ANCOVA) with adjustment for PI, TCI and volume of resting saliva as previous studies [11,27,34] had reported a positive association between these factors and bacterial counts.

For all the analyses, a 5% significance level was used to reject the Null Hypothesis.

### Ethical approval and registration

The Ethical Committee for Human Research at Tokyo Medical and Dental University approved this clinical study (No.238). The trial is registered with ClinicalTrials.gov protocol registration system, ID NCT00748943.

## Results

### Characteristics and oral status of subjects

All 15 subjects completed the study. The oral status of subjects were as follows (mean ± S.D.): mean number of decayed teeth (DT), 2.7 ± 2.0; missing teeth (MT), 0.5 ± 1.4; filled teeth (FT), 5.6 ± 4.2; DMFT, 8.7 ± 5.2 and mean periodontal pocket depth, 2.4 ± 0.5 mm. There were no statistically significant differences in the oral conditions of the subjects in the two groups at the beginning of the study.

### Oral malodor assessments

All of the oral malodor assessments, organoleptic measurement (OM) and gas chromatographic (GC) assessment are listed in Table 1 and show the same general trends. There was no statistically significant difference between the control group and the experimental group at baseline. Statistically significant improvements in reducing oral malodor occurred in the experimental group with ClO_2 _mouthwash used for 7 days, compared with baseline scores (p < 0.01).

**Table 1:** Mean values of OM score, H_2_S, CH_3_SH and (CH_3_)_2_S at baseline and following 7 days rinsing.

		Baseline Mean (SD^a^)	7 days after Mean (SD^a^)	*p*-value^b^
OM^a^	Experimental group	2.10 (0.51)	1.43 (0.46)	0.003**
	Control group	1.87 (0.61)	1.73 (0.56)	0.340
	*p*-value^c^	0.367	0.174	
				
H_2_S^a^(ng/10 mL)	Experimental group	5.31 (4.89)	0.90 (0.93)	0.000**
	Control group	4.88 (6.61)	4.78 (5.90)	0.720
	*p*-value^d^	0.324	0.002**	
				
CH_3_SH^a^(ng/10 mL)	Experimental group	1.42 (1.48)	0.19 (0.29)	0.000**
	Control group	1.21 (1.45)	1.10 (1.14)	0.288
	*p*-value^d^	0.299	0.000**	
				
(CH_3_)_2_S^a^(ng/10 mL)	Experimental group	0.40 (0.27)	0.07 (0.11)	0.000**
	Control group	0.33 (0.33)	0.28 (0.29)	0.567
	*p*-value^d^	0.240	0.048*	

a OM, organoleptic measurement; H_2_S, hydrogen sulfide; CH_3_SH, methyl mercaptan; (CH_3_)_2_S, dimethyl sulfide; SD, standard deviation

b Comparision with the baseline (before rinsing) and after 7 days, OM score was used by the Willcoxson signed-rank test and the log transformed concentrations of H2S, CH3SH and (CH3)2S were used by paired t-test for statistical analysis.

c Comparision with the experimental group and control group on OM. The Mann-Whitney *U*-test was used for statistical analysis.

d Comparision with the experimental group and control group on the log transformed concentrations of H_2_S, CH_3_SH, (CH_3_)_2_S. Independent t-test was used for statistical analysis.

*p < 0.05, **p < 0.01

The placebo mouthwash used for 7 days, on the other hand, showed no statistically significant difference in oral malodor compared with baseline scores.

After rinsing for 7 days, especially, the mean concentrations (ng/10 mL) of H_2_S, CH_3_SH and (CH_3_)_2_S in the experimental group were remarkably reduced compared with those in the control group.

### Oral status evaluations

The results of the PI, GI, TCI, TDI and resting saliva (mL/min) at baseline and after 7 days are shown in Table 2. With the experimental mouthwash used for 7 days, a statistically significant inhibition in plaque accumulation was evident compared with before rinsing (p < 0.05). With the placebo mouthwash used for 7 days on the other hand, no statistically significant inhibition was observed compared with before rinsing. Further, the mean score of the experimental group was not significantly lower than the mean plaque score of the control group after 7 days. Although the mean score of GI reduced with the experimental mouthwash used for 7 days, there was no statistically significant difference compared with the GI value before rinsing.

**Table 2:** Mean scores of PI, GI, TCI, TDI and resting saliva at baseline and following 7 days rinsing.

		Baseline Mean (SD)	7 days after Mean (SD)	*p*-value^b^
PI^a^	Experimental group	1.13 (0.56)	0.92 (0.40)	0.027*
	Control group	1.11 (0.58)	1.15 (0.35)	0.865
	*p*-value^c^	1.000	0.098	
				
GI^a^	Experimental group	1.08 (0.71)	0.90 (0.93)	0.200
	Control group	1.07 (0.73)	1.03 (0.42)	0.649
	*p*-value^c^	1.000	0.539	
				
TCI^a^	Experimental group	9.00 (1.96)	7.07 (1.75)	0.005**
	Control group	9.10 (2.17)	7.13 (1.68)	0.023*
	*p*-value^c^	1.000	0.806	
				
TDI^a^	Experimental group	9.47 (3.09)	8.93 (3.03)	0.632
	Control group	9.45 (3.02)	7.60 (2.50)	0.074
	*p*-value^c^	1.000	0.174	
				
Resting saliva (mL/min)	Experimental group	0.33 (0.20)	0.38 (0.30)	0.196
	Control group	0.43 (0.33)	0.46 (0.36)	0.085
	*p*-value^c^	0.436	0.367	

a PI, plaque index; GI, gingival index; TCI, tongue coating index; TDI, tongue discoloration index

b Comparision with the baseline (before rinsing) and after 7 days. The Willcoxson signed-rank test was used for statistical analysis.

c Comparision with the experimental group and control group. The Mann-Whitney *U*-test was used for statistical analysis.

*p < 0.05, **p < 0.01

With the experimental mouthwash used for 7 days, statistically significant inhibition in tongue coating was evident compared with before rinsing (p < 0.01). For the placebo mouthwash used for 7 days, statistically significant inhibition in tongue coating occurred compared with levels before rinsing (p < 0.05). The TCI scores of subjects within the experimental group were not significantly lower than those of the control group after 7 days. There was no statistically significant difference between the TDI scores and resting saliva volume with either the experimental or placebo mouthwash after 7 days.

### Microbiological results

The results from bacterial counts are shown in Table 3. There were no statistically significant differences between the numbers of total bacterial counts with experimental and the placebo mouthwash used over 7 days. The detection threshold for this method was 10 copies of bacterial DNA per reaction.

**Table 3:** Mean number counts (log_10 _(copies/10 μL)) of selected bacterial species in saliva at baseline and following 7 days rinsing.

		Baseline Mean (SD)	7 days after Mean (SD)	*p*-value^b^
Total bacterial counts	Experimental group	8.33 (0.48)	8.04 (0.55)	0.278
	Control group	8.41 (0.58)	8.21 (0.58)	0.411
	*p*-value^b^	0.734	0.491	
				
*F. nucleatum*^a^	Experimental group	5.09 (0.75)	4.29 (1.05)	0.006**
	Control group	5.07 (0.56)	4.85 (0.65)	0.418
	*p*-value^b^	0.822	0.024*	
				
*P. gingivalis*^a^	Experimental group	1.87 (1.66)	1.75 (1.31)	0.618
	Control group	1.83 (1.52)	1.82 (1.57)	0.796
	*p*-value^b^	0.614	0.866	
				
*T. forsythus*^a^	Experimental group	4.11 (0.87)	3.87 (1.02)	0.443
	Control group	4.01 (0.63)	3.77 (0.70)	0.366
	*p*-value^b^	0.734	0.993	
				
*T. denticola*^a^	Experimental group	2.82 (1.24)	2.86 (1.19)	0.849
	Control group	2.79 (1.29)	2.68 (1.22)	0.797
	*p*-value^b^	0.926	0.676	

a *Fusobacterium nucleatum; Prophyromonas gingivalis; Tannerella forsythensis; Treponema denticola*

b Comparision with the baseline (before rinsing) and after 7 days on the bacterial number counts. Data were analyzed using ANCOVA with adjustment for PI, TCI and volume of resting saliva.

*p < 0.05, **p < 0.01

At baseline, *Fusobacterium nucleatum *(*F.n*.) was detected in all 15 subjects. With the experimental mouthwash used for 7 days, one subject did not detected any *F.n*. and the mean counts of *F.n*. reduced, showing statistically significant bacterial inhibition in saliva compared with the baseline (p < 0.01). The placebo mouthwash group after 7 days, on the other hand, showed no statistically significant inhibition compared with the baseline. There were statistically significant differences between the mean number of *F.n*. counts in the experimental and those in the control group after 7 days (p < 0.05).

With the experimental mouthwash used for 7 days, the mean number of *Prophyromonas gingivalis (P.g.) *count reduced, however, there was no significant difference compared with the before rinsing baseline. In the control group, no statistically significant inhibition was observed compared with the baseline. *P.g*. were detected in four subjects before and after mouth rinsing in both experimental and control group. There was no statistically significant difference between the numbers of *P.g*. counts in the experimental and control groups after 7 days.

At baseline, *Tannerella forsythensis (T.f.) *were detected in all subjects, however, after using the experimental mouthwash for 7 days, it was not detected in one subject. The experimental mouthwash reduced the mean number of *T.f*. counts after 7 days, however, there was no statistically significant difference compared with baseline counts.

There were no statistically significant differences between the numbers of *Treponema denticola (T.d.) *counts with experimental and the placebo mouthwash used over 7 days.

### Subjects perceptions of the mouthwashes

Over the 7-day period, either the experimental or placebo mouthwash was used on 14 occasions. The interval from last rinsing with the experimental mouthwash to assessment on subjects malodor was 8.80 ± 1.53 hours, and 9.23 ± 1.07 hours with the placebo mouthwash. There was no statistically significant difference between the time intervals and examination with the two mouthwashes.

Nine subjects reported a "fresh breath feeling" after rinsing with the experimental mouthwash. On the other hand, only three subjects reported the same feeling with the placebo mouthwash. Eleven subjects perceived they had a "reduced bad breath" after rinsing with the experimental mouthwash, and three subjects reported the same feeling with the placebo mouthwash. With the experimental mouthwash, three subjects reported problems such as "dislike of the smell and taste". With the placebo mouthwash, no subject reported a problem.

## Discussion

In this randomized clinical trial, two mouthwashes were compared; one with ClO_2 _and one without ClO_2_, to investigate the malodor, salivary bacteria and reducing effects of ClO_2_. The results of this study demonstrate that rinsing with a mouthwash containing ClO_2_, used over a 7-day period, was effective in reducing morning oral malodor, plaque, tongue coating accumulation and the counts of *Fusobacterium nucleatum *in saliva in healthy subjects.

Chlorine dioxide (ClO_2_) is a stable free radical. It is readily soluble in water forming a light clear yellow-colored solution in which it can remain intact for considerable periods of time. Oral rinses containing ClO_2 _are now utilized in dental practices as a topical antiseptic for the oral cavity or for dentures [21,35,36]. Previous studies have suggested that ClO_2 _and ClO_2_^- ^are chemically reactive oxidants with powerful reducing capacity on VSCs. Lynch et al. reported that reaction of L-cystein, a thiol compound which contribute substantially towards oral malodor [16], with ClO_2_^. ^and/or ClO_2_^.-^, which contained 0.10% (w/v) ClO_2 _(the same as the experimental mouthwash used in this study), yielded the disulfide cystine as a major product. The processes for the oxidation of thiols through the consecutive, two-step reaction sequence involving ClO_2_^. ^and/or ClO_2_^.- ^are shown as the following equations: (1) RSH (e.g. CH_3_SH) + ClO_2_^. ^→ RS^. ^+ ClO_2_^.-^+ H^+^; (2) 2RS^. ^→ RSSR (e.g. CH_3_SSCH_3_); (3) 4RSH + ClO_2_^.- ^→ 2RSSR + Cl^- ^+ 2H_2_O [17]. Grootveld et al. reported that the oral rinse containing ClO_2 _suppressed saliva numbers of *Streptococcus mutans *and lactobacilli *in vivo*, observed reflecting the bacterocidal action of oxohalogen oxidants present [19]. Though a few subjects reported a problem of a chloric smell, chlorite anion is powerfully bactericidal to malodorous microorganisms [18]. Chlorine dioxide penetrates the bacterial cells and reacts with vital amino acids in the cytoplasm to kill the organism [21,35]. It is reported to exert its bactericidal effects by fixing cellular membrane proteins as a result of its oxidizing potential in a similar manner to oxidizing agents [37].

Several methods have been developed to identify these microorganisms, many of which are polymerase chain reaction (PCR)-based bacterial detection systems [38]. Most of the reported PCR-based diagnostic systems are qualitative analysis methods and are therefore unsuitable for the accurate evaluation of bacteria causing oral malodor [34]. A real-time PCR assay has been developed for the quantitative detection of DNA copy numbers [39]. In this study we used a newly developed Invader PLUS technology. This is a sensitive, rapid method for detection and quantification of nucleic acid. While the original technology is based on the amplification by PCR of the target sequence followed by its detection using the Invader technology, the current modification allows simultaneous PCR amplification and Invader reaction. This allows simpler design and faster results. This technology has been applied for the quantification of periodontitis-related bacteria; *F.n.; P.g.; T.f*., and *T.d*. [29].

VSCs have been shown to result from the bacterial putrefaction of proteins with sulfur-containing amino acids [1]. These proteins are derived from tongue epithelial cells and white blood cell debris [6,7]. Bacteria such as *P g., F.n., T.f., T.d*., and several species of other oral bacteria associated with gingivitis and/or periodontitis are known to produce large amounts of VSCs, which are malodorous. Periodontal disease causes high concentrations of VSCs in mouth air. The concentrations of CH_3_SH are significantly higher in patients with periodontal disease than those in orally healthy individuals [10]. Although the current study was conducted with orally healthy subjects, the results suggest that a mouthwash containing ClO_2 _might reduce bacterial load (as seen in *F.n*. reduction) and lower oral malodor in patients with periodontal disease.

*F.n*. produces both H_2_S and CH_3_SH from the saliva, dorsum of the tongue and sub-gingival plaque [10]. *F.n*. is considered a 'bridge-organism' that facilitates colonization of other periodontal malodorous bacteria especially *T.f*. by coaggregation-mediated mechanisms [40,41]. Moreover it was reported that *F.n *was an important bacterium in the development of complex dental plaque biofilms [42]. Therefore the results of this study suggested that the reducing effects on morning oral malodor, plaque and tongue coating accumulation, was partially caused by reducing the counts of *F. n*. In this study, however, we were unable to find a statistically significant reduction of *P.g., T.f*. and *T.d*. bacterial load using the ClO_2 _mouthwash. Though we found a significant effect on plaque accumulation using the ClO_2 _mouthwash over a 7-day period, this was not translated into a significant inhibitory effect on gingivitis. Previous reports showed that the rinsing by CIO_2 _mouthwash reduced salivary bacteria such as *Streptococcus mutans *and *lactobacilli *[19]. However there is no report about periodontitis-related bacteria. In this study, we demonstrated that 7-day rinsing by CIO_2 _mouthwash reduced oral malodor and only *F.n*. counts in saliva. The prevalence of *F.n*. was highest in saliva and it had shown a high capability of producing VSCs [43]. On the other hand, the prevalence of the periodontal disease-associated bacteria such as *P.g*. and *T.d*. were lower than *F.n*. in saliva of these healthy subjects. Kurata et al. reported that the prevalence of *P.g*., *T.f*. and *T.d*. in saliva was related to periodontal health status and VSC levels in mouth air in patients with periodontal disease, however the prevalence of these bacteria was low in patients without periodontal disease [44]. We need therefore to examine the long-term effects of ClO_2_, on periodontal diseases and microbiological activities, and use a larger sample size in future research.

The finding that there was no difference in the level of tongue coating between the experimental and placebo mouthwash may relate to the mechanism of using both mouthwashes in a 'gargling' fashion. For example, Hakuta et al. reported that 'gargling' with water everyday reduced the tongue coating of the elderly subjects in her study of oral function [45].

Frascella tested the effectiveness of a ClO_2_-containing mouthwash at different points of time for a total of 96 hours after rinsing [23]. The results showed a significant improvement in OM scores and VSC levels measured by a portable sulfide monitor when the tested mouthwash was compared to a water control. The mean VSC concentration in the test group maintained its effective level at 8 hours after rinsing. In the present study, the interval from the last rinsing (before sleeping) with the experimental mouthwash to the assessment of subjects oral malodor was an average 8.80 (range 6 to 11) hours. We found that rinsing with ClO_2 _dramatically reduced the concentrations of all three kinds of VSCs, on the morning of the assessment day. However after the one week washout period, the VSCs level returned to those at the baseline. It is suggested that residual ClO_2 _remaining in the saliva or oral cavity may have reduced VSC level for at least about 9 hours. Further research should define the maximum effective time on VSC reduction and that trials should be conducted over longer time periods, 2-4 weeks or longer.

Recently, many over-the-counter mouthwashes have been used in the treatment of oral malodor. Some of these products merely mask malodor. The optimal mouthwash to treat oral malodor would be an antiseptic agent with proven long-lasting efficacy for reduction of OM and VSC concentrations, with no or few side effects. Chlorhexidine-containing mouthwashes inhibit formation of VSCs and are effective oral antiseptics with antiplaque and antigingivitis effects [46]. Although CHX is considered the most effective oral antiseptic agent, Gürgan et al reported using 0.2% alcohol-free CHX mouthrinse for 1 week caused more irritation to oral mucosa, greater burning sensation, and increased altered taste perception compared to the placebo rinse [14]. Listerine^® ^(Johnson and Johnson, New Jersey, USA), a mouthwash containing essential oils, may also have antiplaque and antigingivitis activity [47]. However, its high alcohol concentration reduces taste sensation and can cause oral pain [48]. Zinc ions inhibit oral malodor but again had a taste problem [49]. Triclosan and cetylpyridium chloride (CPC) are antimicrobial agents widely used as antiseptic agents [50]. However, their clinical reduction of VSCs is questionable [51].

ClO_2 _is used widely in various fields for its safe and high antibacterial action [16]. Sodium chlorite (NaClO_2_), equivalent to ClO_2_, the traditional ingredient in almost all oxygen supplementation today, is a non-toxic substance approved by the U.S. Food and Drug Administration (FDA) as an antimicrobial agent [52]. We found ClO_2 _not only to be effective at reducing oral malodor, but also none of the volunteers complained about tongue stimulation or discoloring with the 0.10% ClO_2 _(0.16% NaClO_2_) mouthwash. For some subjects, the taste and smell of this mouthwash were disagreeable. This may be resolved in new formulations which masks these problems.

Kimoto et al investigated the antibacterial effects of a mouthwash containing ClO_2 _and it's cytotoxicity on human oral cells, for the purpose of using ClO_2 _as a bactericidal agent for natural teeth, dental implants and generally within the oral cavity. Their results suggest that the mouthwash containing ClO_2 _is harmless for human cells and possible to use as a bactericidal agent for dental implants [53]. A proliferation of oral bacteria during sleep is responsible for the release of offending gases, most of which are VSCs. This is often described as "morning bad breath" and occurs even in healthy people [54]. A substantial proportion of healthy people complain of this form of oral malodor. Healthy individuals who suffer from bad breath are likely to use mouthwashes containing several masking or antimicrobial agents. Therefore, recent papers have pointed out the relevance of comparative studies to verify the efficacy of the mouthwashes on "morning bad breath" in healthy subjects [8,55,56]. Most former mouthwash studies used healthy subjects with no complaints about oral malodor, often lacked an adequate control and were evaluated only over a short-term effect of the agent [8,20,55]. Our study also investigated only the short-term effects of the mouthwash in healthy subjects. It is not known therefore whether the same results would be obtained from patients presenting with halitosis as a clinical problem. Future research is needed to examine long-term effects, as well as effects on periodontal diseases and plaque accumulation in a well-defined sample of halitosis patients. It is also recognized that comparative efficacy studies need to be performed against the known effective mouthwashes containing CHX [12,13]. Additional research should also be conducted in broader population samples, including females. Nonetheless, in this explorative study, the OM score was improved and VSCs concentrations were significantly reduced using the ClO_2 _mouthwash. Therefore, the mouthwash clearly demonstrated an anti-malodor effect on morning breath, potentially without any measurable side effects in healthy subjects.

## Conclusion

The results showed that a mouthwash containing ClO_2 _improved morning bad breath measured with the OM and reduced the concentrations of H_2_S, CH_3_SH and (CH_3_)_2_S measured by gas chromatography in healthy subjects. Moreover ClO_2 _mouthwash used over a 7-day period was effective in reducing plaque, tongue coating accumulation and the counts of *Fusobacterium nucleatum *in saliva. However, future studies are needed to examine more long-term effects of the mouthwash in halitosis patients and broader population samples.

## Competing interests

The authors declare that they have no conflict of interests. Materials and support were made available to the authors independent of outcome and consequence.

## Authors' contributions

KS has made substantial contribution to the study conception and design and obtained the ethics approval. KS, CK, ST, SY, TZ and MO implemented this study and participated in the acquisition, analysis and interpretation of data. KS, MU FACW and YK have been intimately involved in drafting and editing the manuscript.
